# Altered N170 and compensatory mechanisms in face processing in schizophrenia: an event-related potential study

**DOI:** 10.3389/fpsyt.2025.1690567

**Published:** 2026-01-12

**Authors:** Jiajun Sun, Qing Liu, Li Liu, Chunhui Bai, Changming Wang, Ling Li, Hongjun Sun

**Affiliations:** 1 School of Psychology and Mental Health, North China University of Science and Technology, Hebei Key Laboratory of Mental Health and Brain Science, Tangshan, Hebei, China; 2 The Third People’s Hospital of Tongliao, Inner Mongolia Autonomous Region, China

**Keywords:** alpha oscillation, event-related potential, face processing, N170, neural compensation, schizophrenia

## Abstract

**Introduction:**

Impaired face processing is a core contributor to social cognitive deficits in schizophrenia. Studies examining the N170, an event-related potential component indexing the structural encoding of faces, have yielded a puzzling inconsistency—showing reduction, no difference, or even enhancement in patients. This paradox challenges simplistic deficit models and may point to uncharacterified compensatory neural mechanisms, potentially arising from dysfunctional neural oscillations that undermine processing efficiency.

**Methods:**

Fifty clinically stable patients with schizophrenia and twenty-five healthy controls performed a challenging perceptual matching task with partially occluded and unoccluded images of faces and buildings during high-density EEG recording. Our analysis specifically targeted the N170 component and its underlying neural synchrony, quantified by the phase-locking factor (PLF) in the alpha band (8–12 Hz). We directly contrasted its response to faces against a control category (buildings) to isolate face-specific processing, and contextualized these findings within the established early visual processing deficit indexed by the P1 component.

**Results:**

We found a significant enhancement of the N170 amplitude specifically to faces in patients versus controls (P = 0.018), with no group difference for buildings (P = 0.846). Critically, this face-specific hyper-responsiveness was accompanied by a significant reduction in alpha-band PLF for the N170 (P = 0.002), indicating impaired precision in neural synchrony despite the larger response. For a comprehensive framework, we also replicated the well-established deficit in early visual processing, as evidenced by a reduced P1 amplitude (P < 0.001).

**Conclusion:**

The combination of an enhanced N170 with impaired alpha-phase locking reveals a hyper-responsive yet inefficient and dyssynchronous face-processing network in schizophrenia. This distinctive electrophysiological profile supports a model of inefficient neural compensation, whereby the brain mobilizes greater resources in an attempt to process socially salient stimuli, but does so with poor temporal coordination, ultimately contributing to the social cognitive impairments characteristic of the disorder.

## Introduction

Schizophrenia is a prevalent, chronic mental disorder that imposes substantial burdens on patients, their families, and society at large (1, 2). Beyond its defining positive and negative symptoms (3), deficits in social cognition—most notably in face perception—are now acknowledged as core features, with strong predictive value for functional outcomes (4). The neurophysiological exploration of these deficits has been greatly advanced by techniques like electroencephalography (EEG) and event-related potentials (ERPs), which deliver millisecond-level temporal resolution to capture dynamic brain processes (5–8). Within this methodological framework, the N170 component has emerged as a key and highly reliable neural marker for the structural encoding of facial stimuli (9).

However, a synthesis of existing literature uncovers a critical, unresolved contradiction: studies report that the N170 in patients with schizophrenia may be reduced, unchanged, or even enhanced relative to healthy controls (10–12). This striking inconsistency challenges the adequacy of a simplistic “deficit model” and points to a more complex pathophysiological basis—one that likely involves dynamic, state-dependent neural processes. We propose that this paradox can be explained by maladaptive compensatory mechanisms: the brain attempts to counteract dysfunction within the core face-processing network, ultimately leading to an altered and potentially inefficient neurophysiological signature. This perspective is supported by a growing body of work emphasizing the role of aberrant neural oscillations in schizophrenia; these oscillations are thought to underlie cognitive deficits by disrupting the temporal coordination of neural assemblies (13).

Building on foundational research that established N170 abnormalities in patients with schizophrenia (14), the present study extends this line of inquiry in two key directions. First, we adopt a face-versus-non-face contrast paradigm to isolate category-specific processing of facial stimuli. Second, and more critically, we move beyond traditional amplitude-based measures of the N170 by integrating fine-grained analyses of neural synchrony—specifically, inter-trial phase consistency (ITPC) and the phase-locking factor (PLF) in the alpha band (8–12 Hz). These metrics are particularly relevant, as alpha oscillations are well-documented to be involved in visual information processing and attentional gating (15). By combining these multi-dimensional indices, our goal is not merely to document N170-related deficits, but to decode the underlying nature of neural dysregulation in schizophrenia. We therefore tested three specific hypotheses derived from an inefficient compensation model: (1) patients would show an enhanced N170 amplitude specifically to faces, but not buildings; (2) this N170 enhancement would be accompanied by a reduced alpha-band phase-locking factor (PLF), indicating impaired neural synchrony; and (3) patients would exhibit a reduced P1 amplitude, confirming early visual processing deficits. This pattern would signature a hyper-responsive yet inefficient face-processing system in schizophrenia.

## Methods

### Participants

Fifty-four outpatients with schizophrenia and thirty-one healthy controls were initially recruited. All patients were clinically stable, met the ICD-10 diagnostic criteria for schizophrenia, and were on a stable regimen of antipsychotic medication. Participant inclusion criteria were as follows: (1) age between 18 and 60 years; (2) normal or corrected-to-normal vision; and (3) right-handedness. Exclusion criteria for all participants included: (1) a history of significant recent life events, substance abuse, or neurological disorders; and (2) inability to fully cooperate with the experimental procedures. Following standard EEG data quality control procedures, which led to the exclusion of four patients and six controls due to excessive artifacts or non-compliance, the final sample consisted of 50 patients and 25 healthy controls. The groups did not differ significantly in gender distribution (X^2^ = 3.145, P = 0.076). Although the patient group was older (patients: 41.73 ± 1.51 years; controls: 28.24 ± 0.61years), a non-parametric test indicated that the overall age distributions were not statistically different (Mann-Whitney U test, H = 0.74, P = 0.82). All participants provided written informed consent, and the study was approved by the Ethics Committee of the Tongliao Mental Health Center.

### Clinical assessments

The symptom severity of patients with schizophrenia was quantitatively assessed using the Chinese Mandarin version of the Positive and Negative Syndrome Scale (PANSS) (16). The PANSS comprises three subscales: a 7-item positive scale, a 7-item negative scale, and a 16-item general psychopathology scale, with each item rated on a 7-point scale. Assessments were conducted by trained raters who had achieved inter-rater reliability. Demographic and clinical data, including illness duration and medication history, were also collected for all participants.

### Stimuli

The visual stimuli consisted of two categories: faces and buildings. A total of 60 neutral face images (30 male, 30 female) were selected from the Chinese Facial Affective Picture System (17). Additionally, 40 images of buildings were selected from a standardized database of architectural images. This category of stimuli was chosen as a control condition because buildings, like faces, are complex visual stimuli with high ecological validity, yet they do not engage the specialized face-processing network. Buildings primarily recruit the parahippocampal place area (PPA), which is involved in processing scenes and spatial layouts, thereby providing a robust contrast to face-selective responses in the fusiform gyrus. This contrast allows us to isolate face-specific processing from general visual or object processing.Moreover, buildings lack the emotional and social salience of faces, minimizing confounding effects of affective processing or prior expertise, which are often compromised in schizophrenia (18). All images were resized to 260×300pixels. To control for low-level features and increase the perceptual demand of the task, a solid color block was used to occlude 50% of the area in each image. All stimuli were presented on a uniform gray background at the center of the screen.

### Experimental design and procedure

**Figure 1:**
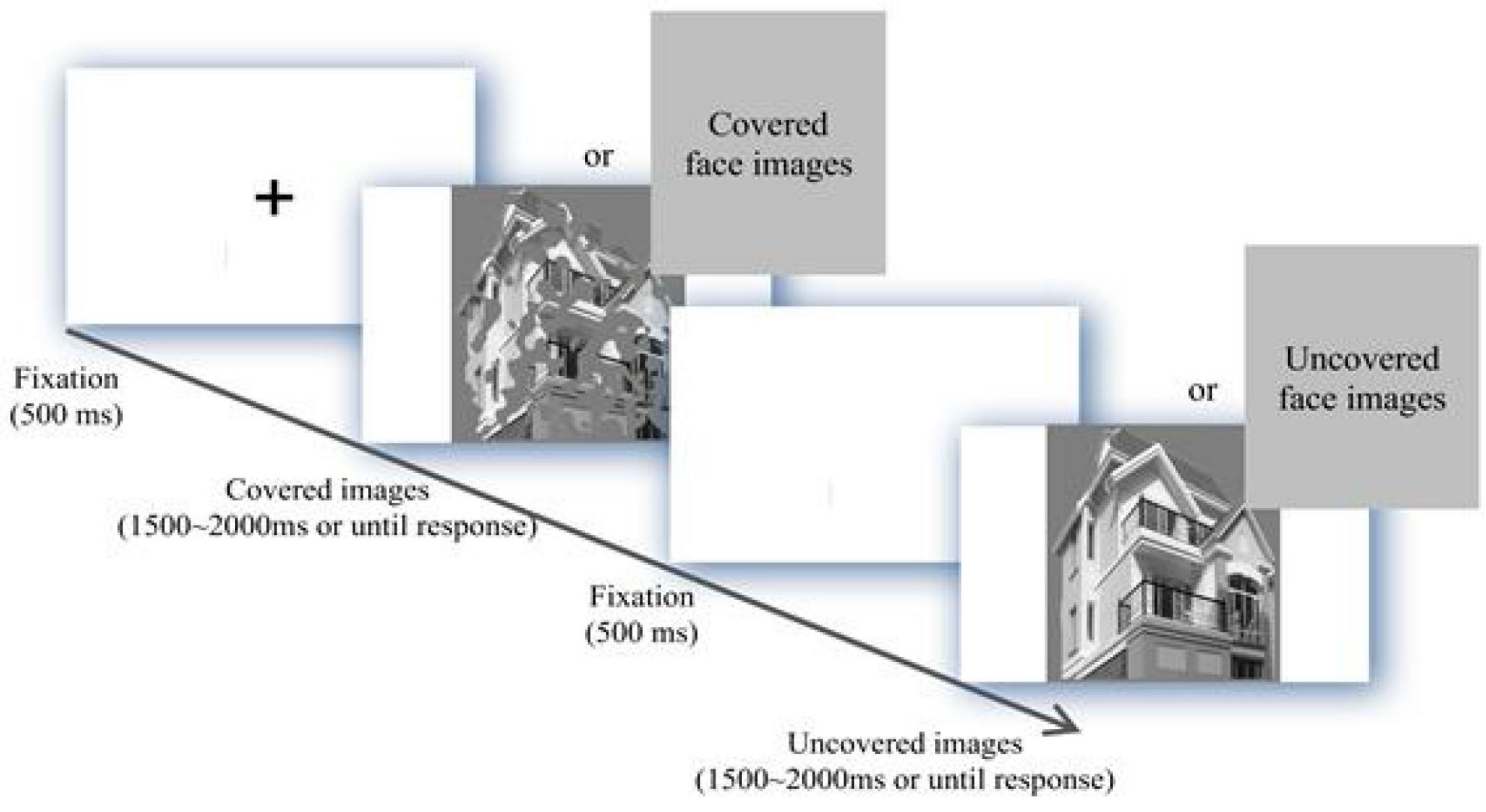
Experimental flow chart.

Participants performed a perceptual matching task where they judged whether sequentially presented pairs of partially occluded and unoccluded images depicted the same identity (faces) or structure (buildings). The trial sequence was: fixation (500 ms), occluded image (1500–2000 ms), fixation (500 ms), unoccluded image (1500–2000 ms). The key manipulation—occluding 50% of the first image—served to degrade low-level input and force reliance on high-order structural encoding mechanisms. This design effectively probed the integrity of face-specific processing (via N170) under challenging conditions against a control condition (building processing), creating a context where compensatory processes might be engaged. The experimental procedure is illustrated in **Figure 1**.

### EEG collection and data processing

This experiment utilized a 32-channel electrode cap based on the international 10–20 system. After placing the electrode cap on the participants, EEG data were recorded using the Neuracle EEG recording system at a sampling rate of 1000 HzBefore data acquisition began, the contact resistance between the electrodes and the scalp was ensured to be less than 30 kΩ. This threshold aligns with the manufacturer’s guidelines for the Neuracle EEG system and is supported by prior clinical studies demonstrating robust ERP acquisition at this level. It represents a balance between signal quality and practical feasibility in a patient population.During the experiment, EEG data were continuously recorded while the participants performed the task.

The EEG data were preprocessed using MATLAB R2018b. Subsequently, batch processing was conducted using the EPAT toolbox developed by Wang Changming (19).

The preprocessing steps were as follows: First, downsampling was performed to reduce the original data frequency to 500 Hz. After downsampling, a bandpass filter with a frequency range of 1.5–30 Hz was applied, and electrode position information was imported. Principal component analysis (PCA) was then employed to identify artifacts caused by eye blinks and eye movements. Next, the data for each participant were reviewed, and segments with significant noise interference were excluded. Regarding parameter settings, the ERP time window was defined as −200 ms before the stimulus to 1.0 second after the stimulus. The data were segmented based on the onset of the unoccluded image stimuli. Segments containing significant noise were excluded, and the remaining trials were averaged to obtain event-related potentials. Finally, all data were re-referenced using the average reference method.

### ERP analysis

In the ERP analysis phase, peak values were extracted within the appropriate time windows to represent the amplitude of each component for each participant. Primary analyses focused on the N170 component (150–190 ms), given its established role in face processing, with additional examination of the P1 (90–130 ms) and P2 (200–270 ms) components.

**Figure 2:**
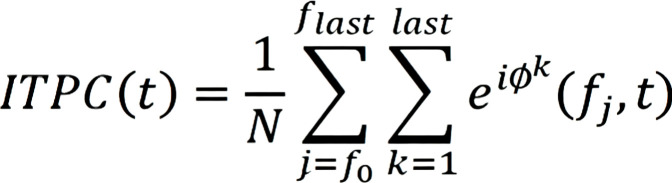
ITPC calculation formula.

Inter-trial phase coherence (ITPC), also known as inter-trial coherence (ITC), was used to measure stimulus-locked evoked activity. ITPC reflects the sum of phase angles across all time segments (see **Figure 2**). ITPC provides a measure of the phase-locking strength between the EEG signal and time-locked events. In this study, the average ITPC value for each ERP component within its respective time window represented the ITPC for that component for each participant.

In the time-frequency analysis, wavelet transform was applied to extract the time-frequency features of the data. Specifically, the preprocessed EEG signals were convolved with complex Morlet wavelets, generating complex wavelet coefficients in the time-frequency domain that contain both amplitude and phase information. The magnitude (i.e., amplitude) of the complex coefficients at each time-frequency point was then calculated, and normalization was performed to standardize the values. Finally, the phase-locking value (PLF), which represents the time-frequency energy distribution of the signal, was obtained. Analyses specifically targeted the alpha band (8–12 Hz) for the P1 and N170 components.

### Statistical analysis

Statistical analysis was performed using SPSS software. Continuous variables that followed a normal distribution were expressed as “mean ± standard deviation.” For inter-group comparisons of ERP component amplitudes and neural synchrony metrics (ITPC, PLF), independent samples t-tests were used. To test for specific interactions between group and stimulus category, repeated-measures analysis of variance was employed where appropriate. Gender distribution differences were assessed using the Chi-square test. A significance threshold of P < 0.05 was applied.

## Results

### Clinical data and scale characteristics

There were no statistically significant differences in age (H = 0.74, P = 0.82) or gender (X^2^ = 3.145, P=0.076) between the schizophrenia group and the healthy control group (see **Table 1**).

**Table 1:** Clinical data and scale characteristics.

Indicator	Schizophrenia group	Normal control group
Age	41.73 ± 1.51	28.24 ± 0.61
Sex ratio (male/female)	40/10	20/5
Duration of illness	6.60 ± 2.72	—
Positive Symptom Scale Total Score	24.92 ± 8.69	—
Negative Symptom Scale Total Score	24.72 ± 8.97	—
General Psychopathology Scale Total Score	56.48 ± 20.79	—

### Component analysis

In this study, components 1, 2, and 3 were identified within three time windows: 90–130 ms, 150–190 ms, and 200–270 ms. The peak amplitude within each specific time window was used to represent the corresponding component. Inter-trial phase coherence (ITPC) was calculated by summing the phase for each trial and averaging it within the time window. Data were analyzed using four electrodes positioned at the forehead,

vertex, and posterior brain regions (**Table 2**). The results revealed that the amplitude and ITPC of component 1 (corresponding to the P1 component in ERP) were reduced in multiple brain regions, particularly in the posterior parietal area (electrodes PO3 and PO4). This suggests that posterior brain regions may play a pivotal role in the pathogenesis of schizophrenia. Consequently, further analysis focused on the data from the PO4 electrode.

**Table 2:** The peak amplitude and ITPC value of each component in the schizophrenia group and the normal group.

Fz	Component & Indicator		
Electrode Site	Schizophrenia group	Normal control group	P
Component 1 (Amplitude)	0.570μV	0.038μV	0.067
Component 1 (ITPC)	0.07	0.135	0.02
Component 2 (Amplitude)	-0.338μV	-0.370μV	0.924
Component 2 (ITPC)	0.162	0.168	0.851
Component 3 (Amplitude)	0.526μV	0.441μV	0.799
Component 3 (ITPC)	0.131	0.128	0.866
Cz			
Component 1 (Amplitude)	0.185μV	-0.013μV	0.343
Component 1 (ITPC)	0.073	0.161	0.004
Component 2 (Amplitude)	-0.179μV	-0.461μV	0.306
Component 2 (ITPC)	0.161	0.167	0.825
Component 3 (Amplitude)	0.739μV	0.491μV	0.383
Component 3 (ITPC)	0.118	0.12	0.92
PO3			
Component 1 (Amplitude)	1.363μV	2.482μV	0.009
Component 1 (ITPC)	0.1	0.182	0.004
Component 2 (Amplitude)	-2.805μV	-2.306μV	0.351
Component 2 (ITPC)	0.212	0.215	0.923
Component 3 (Amplitude)	2.544μV	2.876μV	0.452
Component 3 (ITPC)	0.195	0.189	0.851
PO4			
Component 1 (Amplitude)	1.523μV	3.047μV	0.001
Component 1 (ITPC)	0.117	0.232	0.004
Component 2 (Amplitude)	-3.361μV	-2.381μV	0.078
Component 2 (ITPC)	0.244	0.247	0.925
Component 3 (Amplitude)	2.732μV	2.467μV	0.555
Component 3 (ITPC)	0.224	0.198	0.453

### General ERP alterations and early visual processing deficits

**Figure 3:**
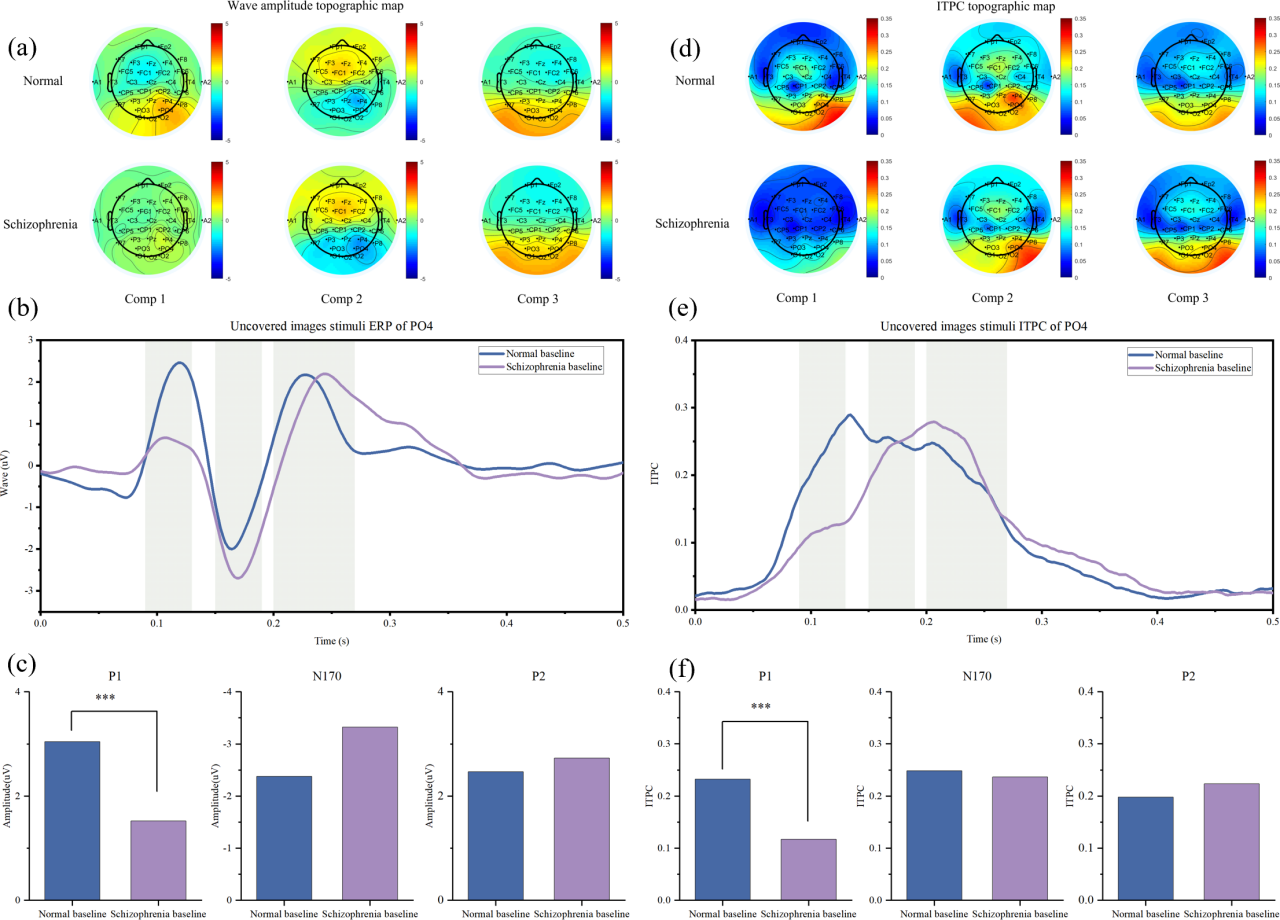
ERP under the condition of uncovered image stimulation. **(a)** Brain topography maps of ERP amplitudes for the 1st group (90–130 ms), 2nd group (150–190 ms), and 3rd group (200–270 ms) comparing the normal and schizophrenia groups. **(b)** ERP waveforms comparing the normal and schizophrenia groups. **(c)** Amplitude comparison of P1, N170, and P2 between the normal and schizophrenia groups. **(d)** ITPC brain topography maps for the 1st group (90–130 ms), 2nd group (150–190 ms), and 3rd group (200–270 ms) comparing the normal and schizophrenia groups. **(e)** ITPC waveforms comparing the normal and schizophrenia groups. **(f)** Comparison of P1, N170, and P2 ITPC between the normal and schizophrenia groups.

The initial component-based analysis revealed widespread abnormalities in the schizophrenia group across early and mid-latency ERP components. As comprehensively visualized in **Figure 3**, patients exhibited a distinct pattern of neural dysfunction. Specifically, the amplitude and inter-trial phase coherence (ITPC) of the P1 component (90–130 ms) were significantly reduced over posterior brain regions, most prominently at the PO4 electrode (amplitude: t =3.55, P < 0.001, **Figure 3c**; ITPC: t=3.04, P = 0.004, **Figure 3f**). The topographic maps further illustrate the diminished and less coordinated neural activity during this early stage in patients (**Figures 3a, d**). This robustly confirms a fundamental, low-level impairment in early visual processing within the dorsal stream, providing a critical physiological context for the subsequent, more specific alterations.

### A face-specific paradox: enhanced N170 amplitude

**Figure 4:**
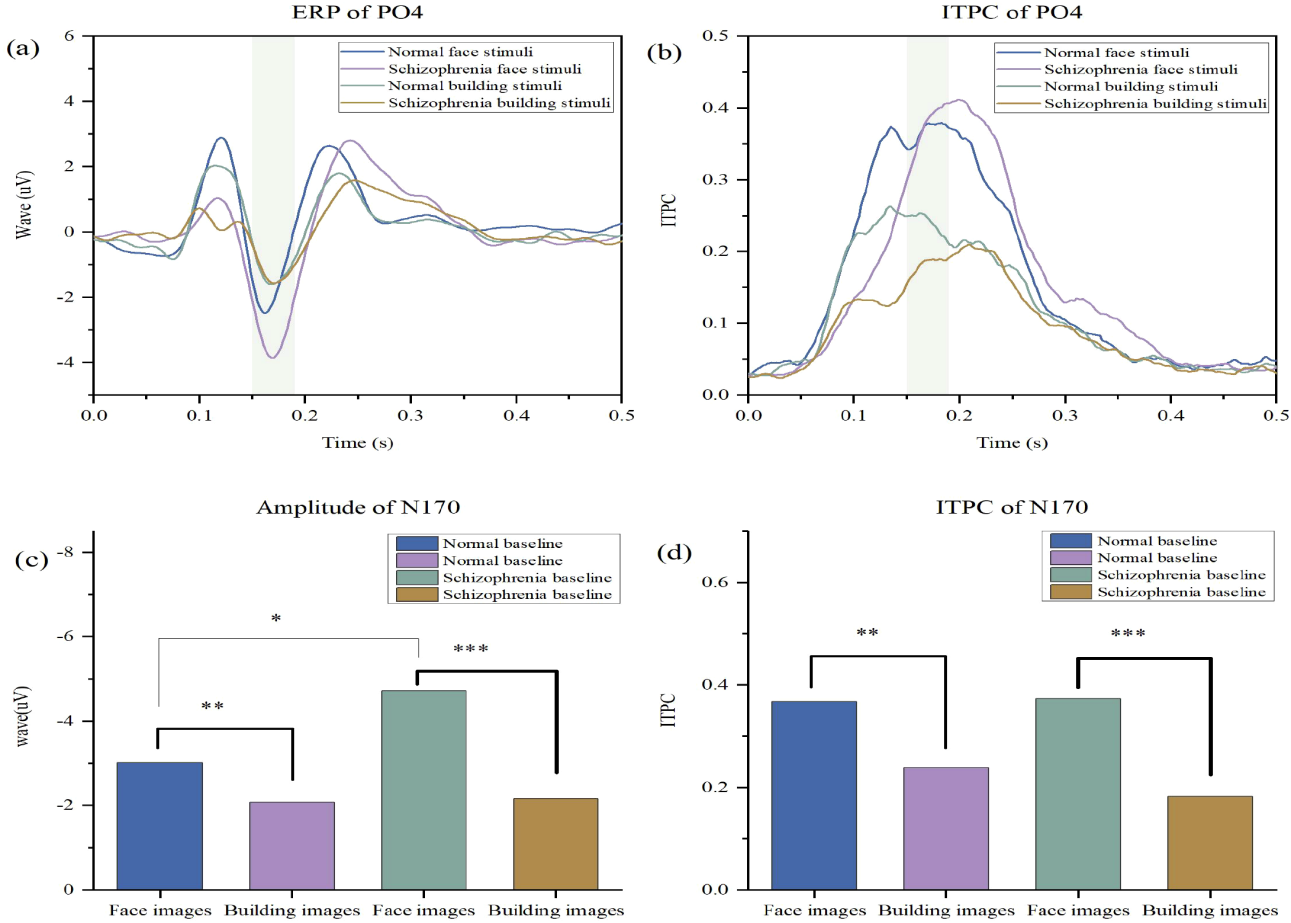
N170 Component Analysis. **(a)** Comparison of N170 waveforms between the normal and schizophrenia groups under unoccluded facial image stimuli and unoccluded building image stimuli. **(b)** Comparison of N170 ITPC between the normal and schizophrenia groups under unoccluded facial image stimuli and unoccluded building image stimuli.n **(c)** Comparison of N170 amplitudes between the normal and schizophrenia groups under unoccluded facial image stimuli and unoccluded building image stimuli. **(d)** Comparison of N170 ITPC between the normal and schizophrenia groups under unoccluded facial image stimuli and unoccluded building image stimuli. (* P < 0.05, ** P < 0.01, *** P < 0.001.).

Building on the general profile of early deficits, a critical and specific alteration emerged when we directly contrasted neural responses to faces and buildings. The N170 component, a key marker of structural face encoding, exhibited a paradoxical profile in patients. The central finding was a marked enhancement of the N170 amplitude in patients with schizophrenia specifically in response to faces compared to healthy controls (t=2.16, P = 0.018, **Figure 4c**). In stark contrast, the two groups showed no significant difference in N170 amplitude when viewing building stimuli (t=0.19, P = 0.846, **Figure 4c**). This dissociative pattern, clearly depicted in the waveform comparisons (**Figure 4a**), indicates that the patients’ neural systems not only preserve the categorical distinction between faces and objects but indeed hyper-accentuate it.

### Impaired neural synchrony underlying the enhanced N170

This paradoxica lN170 hyper-responsiveness to faces was accompanied by a significant reduction in the precision of neural synchrony. Time-frequency analysis revealed a substantial decrease in the phase-locking factor (PLF) within the alpha band (8–12 Hz) for the N170 component in patients (P = 0.002). The topographic distribution of this alpha-band PLF was also more widespread and diffuse in the schizophrenia group (**Figure 4b**), suggesting that the hyper-responsive face processing is subserved by a less focal and less coordinated neural computation.

### Stimulus-specific alterations in neural oscillations

Supporting the component-specific findings, a generalized analysis of neural oscillations further delineated the nature of the deficit. The schizophrenia group demonstrated significantly lower alpha-band PLF values compared to controls for both facial (t = 2.041, P = 0.045) and building images (t = 3.812, P < 0.001). Notably, the neural contrast in PLF between face and building stimuli was itself markedly greater in the schizophrenia group (t = 4.825, P < 0.001) than in controls (t = 2.452, P = 0.018) (**Figure 4d**). This exaggerated differential response further reflects a dysregulated allocation of neural resources.

### An integrated pathophysiological model

**Figure 5:**
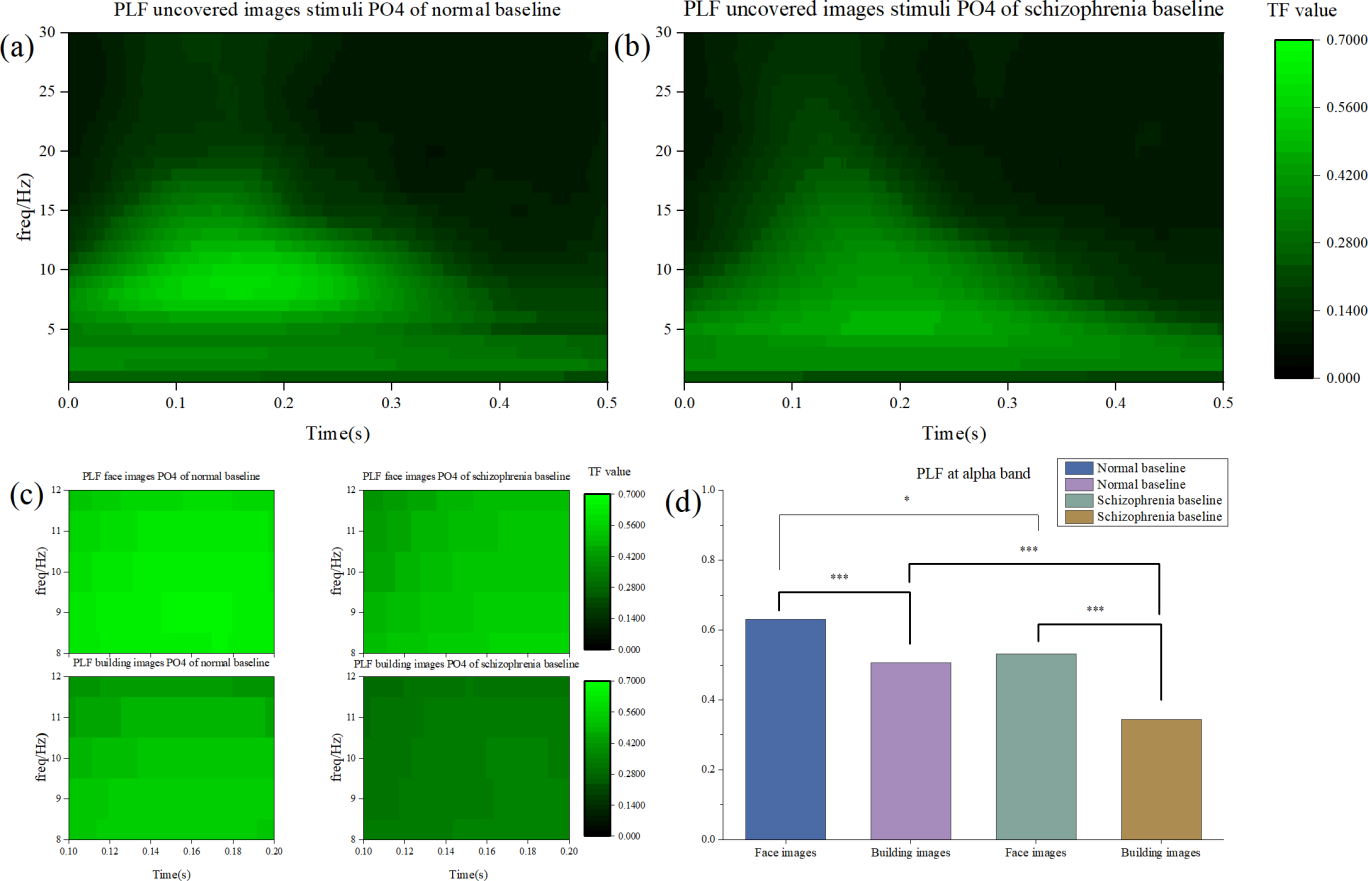
Time-frequency analysis. **(a)** PLF values of the normal group under unoccluded visual stimulus conditions. **(b)** PLF values of the schizophrenia group under unoccluded image stimulus conditions. **(c)** PLF values in the α frequency band. Top left: PLF values of the normal group under unoccluded facial image stimulus conditions. Top right: PLF values of the schizophrenia group under unoccluded facial image stimulus conditions. Bottom left: PLF values of the normal group under unoccluded building image stimulus conditions. Bottom right: PLF values of the schizophrenia group under unoccluded building image stimulus conditions. **(d)** Comparison of α band PLF between the normal group and the schizophrenia group under unoccluded facial image and unoccluded building image stimulus conditions.

To synthesize these multifaceted findings—the early dorsal stream deficit (P1), the face-specific ventral stream hyper-responsiveness (N170 amplitude), and the pervasive impairment in neural synchrony (ITPC/PLF)—we propose an integrated pathophysiological model, which is schematically illustrated in **Figure 5**. This model posits that structural and functional deficits in early visual processing create a degraded input to the core face-processing network. In response, the brain engages an inefficient compensatory mechanism, recruiting a broader and less specialized neural ensemble. This leads to the observed electrophysiological signature:a hyper-active yet dyssynchronous and inefficient response to socially salient stimuli, ultimately contributing to the social cognitive impairments in schizophrenia.

## Discussion

This study provides compelling electrophysiological evidence for a paradoxical and inefficient compensatory mechanism within the face-processing network of individuals with schizophrenia. We replicated the well-documented deficit in early visual processing—evidenced by reduced P1 amplitude and inter-trial phase coherence (ITPC) (**Figure 3**)—a finding consistent with established literature on early visual pathway abnormalities in schizophrenia (20). Our central and novel finding, however, lies in the face-specific enhancement of N170 amplitude, which co-occurs with a marked reduction in its alpha-band phase locking (**Figure 4**). This pattern of hyper-responsiveness coupled with hypo-synchrony challenges the simplistic “deficit-only model,” instead pointing to a more complex, maladaptive compensatory process (21).

The face-specific N170 amplitude enhancement indicates that the core face-processing system in schizophrenia is not merely impaired, but exhibits exaggerated neural responses to socially salient stimuli. This likely reflects the system’s attempt to extract additional information from facial inputs, driven by noisy or deficient signals from earlier processing stages (20). Critically, however, this heightened responsiveness does not translate to improved neural efficiency. On the contrary, the significant reduction in alpha-band phase-locking factor (PLF) and its more widespread topographic distribution in patients reveal a fundamental disruption in the temporal coordination of underlying neural assemblies. This observation aligns with recent work demonstrating impaired alpha-band synchronization during visual processing in schizophrenia (22). In essence, the brain mobilizes a broader, less specialized network to support face processing—achieving greater amplitude at the cost of oscillatory stability and processing precision.

This interpretation aligns with and extends prior research. For instance, while Jung et al. (10) reported reduced N170 current source density in response to emotional faces, our findings suggest the system’s responses are not uniformly diminished. Instead, they can be paradoxically enhanced under certain conditions—likely fueled by compensatory efforts—a pattern consistent with the variable N170 profiles reported in recent meta-analyses (23). The key advancement here is the combined assessment of amplitude and neural synchrony, which uncovers that a response appearing “superior” (i.e., larger amplitude) is in fact underpinned by less efficient neural computation.

Our proposed model—summarized in **Figure 5**—integrates these findings into a cohesive pathophysiological account. It posits that structural and functional deficits in early visual processing (indexed by reduced P1) result in degraded input to the core face-processing network (24). In response, the ventral stream activates an inefficient compensatory mechanism, recruiting a broader neural ensemble (25). This gives rise to the signature pattern observed: a hyperactive yet dyssynchronous and inefficient response to faces. This model provides a unifying framework for interpreting the variable N170 profiles in the literature, linking them to a dynamic imbalance between functional demand and neural resource allocation.

The observed deficits in phase synchronization (ITPC and PLF) further highlight a core pathophysiological mechanism in schizophrenia: impaired neural timing. As suggested by neuropathological studies (26), this dyssynchrony may stem from white matter abnormalities or aberrant myelination—disrupting the precise temporal coordination required for efficient interregional information transfer. Such timing deficits have been specifically linked to abnormal alpha oscillations in schizophrenia (27), thereby weakening the functional connectivity essential for intact visual and social cognition.

In conclusion, perceptual and social cognitive impairments in schizophrenia may arise not only from localized deficits but also from the failure to efficiently integrate neural activity across distributed networks. The identified electrophysiological indices—particularly the dissociated N170 profile—offer a potential biomarker for assessing the integrity of face-processing circuits and the compensatory strategies they employ. Future research should validate this model in larger, age-matched cohorts and explore whether neuromodulation techniques targeting alpha-band oscillations can stabilize neural synchrony and improve processing efficiency—potentially opening new avenues for cognitive remediation in schizophrenia.

### Limitation

This study involved a considerable age difference between the two sample groups, and the control group had a relatively small sample size, which may impact the results. Future research should aim to expand the sample size, with particular focus on clinical control groups, as well as anatomical studies and biomedical imaging observations, to validate the structural damage theory and confirm that structural damage in the nervous system is the underlying cause of the observed electrophysiological changes.

## Conclusion

This study reveals a novel electrophysiological signature in schizophrenia: a face-specific enhancement of the N170 amplitude coupled with a significant reduction in alpha-band phase synchronization. This paradoxical profile challenges the conventional deficit model and is best explained by an inefficient compensatory mechanism, wherein the face-processing network hyper-engages, yet does so in a desynchronized and functionally disorganized manner.The dissociation between the compromised early processing (P1) and the hyper-responsive mid-latency encoding (N170) underscores distinct pathophysiological processes within the visual streams. These findings provide a new framework for understanding social cognitive deficits in schizophrenia, suggesting they may arise not from a simple loss of function, but from a faulty and resource-intensive attempt at compensation.The identified indices—particularly the dissociated N170 profile—offer objective biomarkers for identifying this compensatory state. Furthermore, the specific impairment in alpha synchrony highlights a potential target for neuromodulation therapies aimed at restoring neural timing and improving computational efficiency, thereby paving the way for more targeted interventions.

## Data Availability

The datasets presented in this article are not readily available because The dataset is available for academic research purposes only. Researchers are required to contact the corresponding author for access approval and must adhere to ethical guidelines, especially regarding data anonymization and proper citation of this study if the data is used. Commercial use or redistribution without written consent is strictly prohibited. Requests to access the datasets should be directed to 1993744064@qq.com.

## References

[1] Huang Y Wang Y Wang H Liu Z Yu X Yan J . Prevalence of mental disorders in China: a cross-sectional epidemiological study. Lancet Psychiatry. (2019) 6:211–24. doi: 10.1016/S2215-0366(18)30511-X 30792114

[2] Solmi M Seitidis G Mavridis D Correll CU Dragioti E Guimond S . Incidence, prevalence, and global burden of schizophrenia - data, with critical appraisal, from the Global Burden of Disease (GBD) 2019. Mol Psychiatry. (2023) 28:5319–27. doi: 10.1038/s41380-023-02138-4 37500825

[3] McCutcheon RA Keefe RSE McGuire PK . Cognitive impairment in schizophrenia: aetiology, pathophysiology, and treatment. Mol Psychiatry. (2023) 28:1902–18. doi: 10.1038/s41380-023-01984-6 PMC10575791 36690793

[4] Green MF Horan WP Lee J . Social cognition in schizophrenia. Nat Rev Neurosci. (2015) 16:620–31. doi: 10.1038/nrn4005 26373471

[5] Abram SV Roach BJ Holroyd CB Paulus MP Ford JM Mathalon DH . Reward processing electrophysiology in schizophrenia: Effects of age and illness phase. NeuroImage Clin. (2020) 28:102492. doi: 10.1016/j.nicl.2020.102492 33395983 PMC7695886

[6] Prasuhn J Kanel P . Editorial: Neuroimaging in psychiatry 2023: neurodegenerative disorders. Front Psychiatry. (2025) 16:1653069. doi: 10.3389/fpsyt.2025.1653069 40704034 PMC12283564

[7] Hamilton A Northoff G . Abnormal ERPs and brain dynamics mediate basic self disturbance in schizophrenia: A review of EEG and MEG studies. Front Psychiatry. (2021) 12:642469. doi: 10.3389/fpsyt.2021.642469 33912085 PMC8072007

[8] Earls HA Curran T Mittal V . A meta-analytic review of auditory event-related potential components as endophenotypes for schizophrenia: perspectives from first-degree relatives. Schizophr Bull. (2016) 42:1504–16. doi: 10.1093/schbul/sbw047 PMC5049529 27217271

[9] Gao C Conte S Richards JE Xie W Hanayik T . The neural sources of N170: Understanding timing of activation in face-selective areas. Psychophysiology. (2019) 56:e13336. doi: 10.1111/psyp.13336 30710345 PMC6508977

[10] Turetsky BI Kohler CG Indersmitten T Bhati MT Charbonnier D Gur RC . Facial emotion recognition in schizophrenia: when and why does it go awry? Schizophr Res. (2007) 94:253–63. doi: 10.1016/j.schres.2007.05.001 PMC2571079 17583481

[11] Maher S Mashhoon Y Ekstrom T Lukas S Chen Y . Deficient cortical face-sensitive N170 responses and basic visual processing in schizophrenia. Schizophr Res. (2016) 170:87–94. doi: 10.1016/j.schres.2015.12.005 26690888 PMC4707115

[12] Curzietti M Chaillou AC Bonnefond A Vidailhet P Doignon-Camus N . Visual expertise for print in schizophrenia: Analysis of the N170 component. Int J Psychophysiol. (2018) 133:111–9. doi: 10.1016/j.ijpsycho.2018.08.001 30092244

[13] Uhlhaas PJ Singer W . Abnormal neural oscillations and synchrony in schizophrenia. Nat Rev Neurosci. (2010) 11:100–13. doi: 10.1038/nrn2774 20087360

[14] Kim DW Shim M Song MJ Im CH Lee SH . Early visual processing deficits in patients with schizophrenia during spatial frequency-dependent facial affect processing. Schizophr Res. (2015) 161:314–21. doi: 10.1016/j.schres.2014.12.020 25553978

[15] Jensen O Mazaheri A . Shaping functional architecture by oscillatory alpha activity: gating by inhibition. Front Hum Neurosci. (2010) 4:186. doi: 10.3389/fnhum.2010.00186 21119777 PMC2990626

[16] Wu BJ Lan TH Hu TM Lee SM Liou JY . Validation of a five-factor model of a Chinese Mandarin version of the Positive and Negative Syndrome Scale (CMV-PANSS) in a sample of 813 schizophrenia patients. Schizophr Res. (2015) 169:489–90. doi: 10.1016/j.schres.2015.09.011 26443481

[17] Gong X Huang Y Wang Y . Revision of the Chinese Facial Expression Picture System. Chin. Ment. Health J. (2011) 25:40–46.

[18] Epstein R Kanwisher N . A cortical representation of the local visual environment. Nature. (1998) 392:598–601. doi: 10.1038/33402 9560155

[19] Shi J Gong X Song Z Xie W Yang Y Sun X . EPAT: a user-friendly MATLAB toolbox for EEG/ERP data processing and analysis. Front Neuroinform. (2024) 18:1384250. doi: 10.3389/fninf.2024.1384250 38812743 PMC11133744

[20] Butler PD Javitt DC . Early-stage visual processing deficits in schizophrenia. Curr Opin Psychiatry. (2005) 18:151–7. doi: 10.1097/00001504-200503000-00008 PMC1994776 16639168

[21] Ford JM Krystal JH Mathalon DH . Neural synchrony in schizophrenia: from networks to new treatments. Schizophr Bull. (2007) 33:848–52. doi: 10.1093/schbul/sbm062 PMC2632315 17567628

[22] Javitt DC Freedman R . Sensory processing dysfunction in the personal experience and neuronal machinery of schizophrenia. Am J Psychiatry. (2015) 172:17–31. doi: 10.1176/appi.ajp.2014.13121691 25553496 PMC4501403

[23] McCleery A Lee J Joshi A Wynn JK Hellemann GS Green MF . Meta-analysis of face processing event-related potentials in schizophrenia. BiolPsychiatry. (2015) 77:116–26. doi: 10.1016/j.biopsych.2014.04.015 24923618

[24] Bergson Z Ahmed AO Bell J Butler PD Gordon J Seitz AR . Visual remediation of contrast processing impairments in schizophrenia: A preliminary clinical trial. Schizophr Res. (2024) 274:396–405. doi: 10.1016/j.schres.2024.10.010 39481234 PMC11620924

[25] Green MF Lee J Wynn JK Mathis KI . Visual masking in schizophrenia: overview and theoretical implications. Schizophr Bull. (2011) 37:700–8. doi: 10.1093/schbul/sbr051 PMC3122285 21606322

[26] Chavarria-Siles I White T De Leeuw C Goudriaan A Lips E Ehrlich S . Myelination-related genes are associated with decreased white matter integrity in schizophrenia. Eur J Hum Genet. (2016) 24:381–6. doi: 10.1038/ejhg.2015.120 PMC4757770 26014434

[27] Hamm JP Peterka DS Gogos JA Yuste R . Altered cortical ensembles in mouse models of schizophrenia. Neuron. (2017) 94:153–167.e8. doi: 10.1016/j.neuron.2017.03.019 28384469 PMC5394986

