# Correction: Altered N170 and compensatory mechanisms in face processing in schizophrenia: an event-related potential study

**DOI:** 10.3389/fpsyt.2026.1870273

**Published:** 2026-06-24

**Authors:** Jiajun Sun, Qing Liu, Li Liu, Chunhui Bai, Changming Wang, Ling Li, Hongjun Sun

**Affiliations:** 1 School of Psychology and Mental Health, North China University of Science and Technology, Hebei Key Laboratory of Mental Health and Brain Science, Tangshan, Hebei, China; 2 The Third People’s Hospital of Tongliao, Inner Mongolia Autonomous Region, China

**Keywords:** alpha oscillation, event-related potential, face processing, N170, neural compensation, schizophrenia

The original author list was:

“Jiajun Sun1, Qing Liu1, Hongjun Sun2, Li Liu2, Chunhui Bai2, Changming Wang1* and Ling Li1*”.

The corrected author list now reads:

“Jiajun Sun¹†, Qing Liu¹†, Li Liu¹†, Chunhui Bai², Changming Wang²*, Ling Li¹* and Hongjun Sun²*”.

A mark has been added next to the names of Jiajun Sun, Qing Liu, and Li Liu to indicate they share first authorship. This appears as follows:

† These authors have contributed equally to this work and share first authorship

Hongjun Sun has been added as a third corresponding author, with email sun4412@sina.com.

The original version of this article has been updated.

